# Supplementary material for: Physical Mechanisms of Intermolecular Interactions and Cross-Space Charge Transfer in Two-Photon BDBT-TCNB Co-Crystals
Source: Nanomaterials (Basel). 2022 Aug 11;12(16):2757. doi: 10.3390/nano12162757 (PMC9414930; doi:10.3390/nano12162757)
Supplement: Supplementary file 1 [file nanomaterials-12-02757-s001.zip › nanomaterials-1846771-supplementary.pdf]

Supplementary Materials

Table S1. Real space functions of BCPs.

a.u.	Density of All Electrons	Lagrangian Kinetic Energy G(r)	Hamiltonian Kinetic Energy K(r)	Potential Energy Density V(r)	Energy Density E(r) or H(r)	Laplacian of Electron Density	Electron Localization Function (ELF)	Localized Orbital Locator (LOL)
1(C-C)	0.0051063485	0.0029673956	−0.0007859007	−0.0021814949	0.0007859007	0.0150131850	0.0208770678	0.1277903658
2(C-C)	0.0064904186	0.0037072615	−0.0008295938	−0.0028776677	0.0008295938	0.0181474214	0.0295290299	0.1488676420
3(C-C)	0.0061834077	0.0035426616	−0.0007827635	−0.0027598981	0.0007827635	0.0173017001	0.0275624305	0.1444442839
4(C-C)	0.0051872817	0.0031608872	−0.0008222022	−0.0023386850	0.0008222022	0.0159323578	0.0194262343	0.1237272293
5(C-N)	0.0039838368	0.0025539379	−0.0005225353	−0.0020314026	0.0005225353	0.0123058928	0.0124137315	0.1011672008
6(S-C)	0.0080338596	0.0047167471	−0.0011163915	−0.0036003557	0.0011163915	0.0233325543	0.0369058586	0.1639985766
7(H-N)	0.0055498658	0.0037842226	−0.0009242861	−0.0028599365	0.0009242861	0.0188340347	0.0170361212	0.1166051129
8(S-N)	0.0022483997	0.0015370029	−0.0005474467	−0.0009895563	0.0005474467	0.0083377983	0.0051032245	0.0672388411
9(N-C)	0.0040950627	0.0025698333	−0.0005516093	−0.0020182241	0.0005516093	0.0124857703	0.0134261652	0.1048338511
10(C-C)	0.0052741821	0.0031747771	−0.0007402748	−0.0024345022	0.0007402748	0.0156602075	0.0203348288	0.1262761722
11(C-C)	0.0058876388	0.0035207265	−0.0008436922	−0.0026770343	0.0008436922	0.0174576748	0.0237915168	0.1353644579
12(C-C)	0.0059158507	0.0035353505	−0.0009506947	−0.0025846558	0.0009506947	0.0179441808	0.0239702506	0.1358124179
13(C-C)	0.0066310608	0.0038859740	−0.0008666705	−0.0030193035	0.0008666705	0.0190105777	0.0288923254	0.1474351796
14(C-N)	0.0020285369	0.0013248710	−0.0004059123	−0.0009189587	0.0004059123	0.0069231335	0.0048650081	0.0658113893
15(H-N)	0.0096469294	0.0071935818	−0.0013340326	−0.0058595492	0.0013340326	0.0341104574	0.0294677624	0.1485670081
16(N-H)	0.0044046580	0.0030335927	−0.0008317821	−0.0022018106	0.0008317821	0.0154614994	0.0123128193	0.1007361698
17(H-N)	0.0032407967	0.0022647694	−0.0006918486	−0.0015729208	0.0006918486	0.0118264720	0.0079609307	0.0825493462
18(N-H)	0.0027702573	0.0019971766	−0.0006535926	−0.0013435840	0.0006535926	0.0106030769	0.0060727807	0.0728353930

Table S2. Numerical values for the energy decomposition of the four dimers.

kcal/mol	Dimer 1	Dimer 2	Dimer 3	Dimer 4
Electrostatics	−10.3 (28.5%)	−0.2 (8.3%)	−10.5 (28.7%)	−2.5 (39.1%)
Exchange	18.6	1.2	18.0	3.3
Induction	−4.0 (11.1%)	−0.3 (12.5%)	−3.5 (9.6%)	−0.6 (9.4%)
Dispersion	−21.8 (60.4%)	−1.9 (79.2%)	−22.6 (61.7%)	−3.3 (51.5%)
Total	−17.5	−1.2	−18.6	−3.1

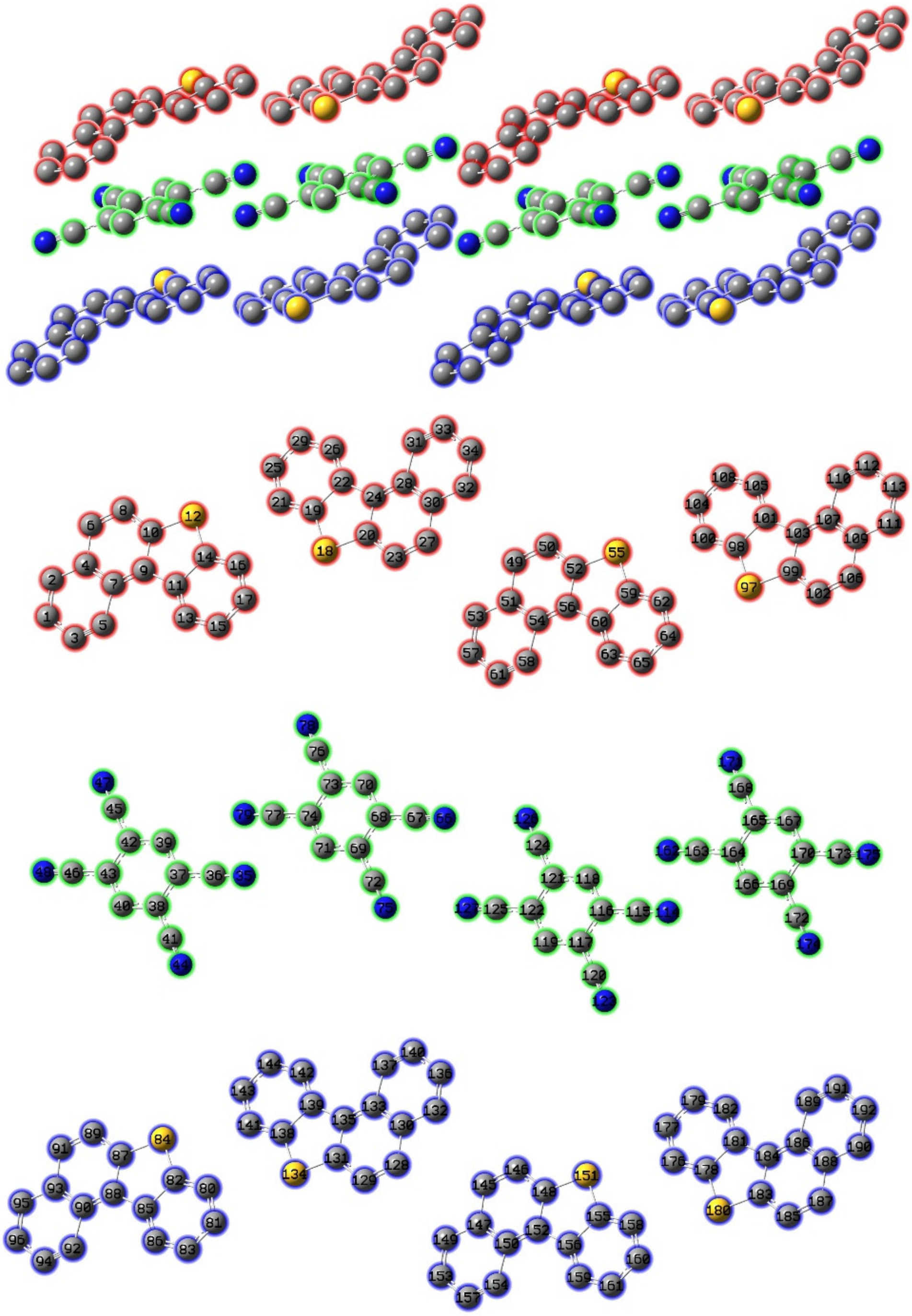

Figure S1. BTC's atomic number.
